# Iron Metabolism, Calcium, Magnesium and Trace Elements: A Review

**DOI:** 10.1007/s12011-024-04289-z

**Published:** 2024-07-06

**Authors:** Tara Rolić, Mazyar Yazdani, Sanja Mandić, Sonia Distante

**Affiliations:** 1 Faculty of Medicine, University of Osijek, Osijek, Croatia; 2 Osijek University Hospital Centre (Klinički bolnički centar Osijek), Osijek, Croatia; 3 Oslo University Hospital, Oslo, Norway

## Abstract

Iron (Fe) is fundamental to life on earth. In the human body, it is both essential and harmful if above threshold. A similar balance applies to other elements: calcium (Ca), magnesium (Mg), and trace elements including copper (Cu), zinc (Zn), lead (Pb), cadmium (Cd), mercury (Hg), and nickel (Ni). These elements share some proteins involved in the absorption and transport of Fe. Cu and Cd can inhibit Fe absorption, while excess of Fe may antagonize Cu metabolism and reduce ceruloplasmin (Cp). Excessive Fe can hinder Zn absorption and transferrin (Trf) can bind to both Zn and Ni. Ca is able to inhibit the divalent metal transporter 1 (DMT1) in a dose-dependent manner to reduce Fe absorption and low Mg concentrations can exacerbate Fe deficiency. Pb competitively inhibits Fe distribution and elevated Cd absorption reduces Fe uptake. Exposure to Hg is associated with higher ferritin concentrations and Ni alters intracellular Fe metabolism. Fe removal by phlebotomy in hemochromatosis patients has shown to increase the levels of Cd and Pb and alter the concentrations of trace elements in some types of anemia. Yet, the effects of chronic exposure of most trace elements remain poorly understood.

## Introduction

Iron (Fe) is abundant in the human body and is a vital element for sustaining life. Its function spans across various biological processes, from oxygen transport in cells to aiding DNA synthesis [1]. Maintaining a precise balance in Fe metabolism is essential for human well-being, necessitating regulation at multiple levels. Central to this regulatory framework is intestinal Fe absorption which is tightly controlled by a number of proteins such as divalent metal transporter 1 (DMT1), ferroportin (FPN1), transferrin (Trf), and transferrin receptors (TrfR). Ferroxidases such as duodenal cytochrome B (DCYTB), ceruloplasmin (Cp), and hephaestin are coordinated by hepcidin (Hep) to ensure efficient Fe acquisition and utilization. As one dives deeper into Fe metabolism, the intricate relationships between Fe, calcium (Ca), magnesium (Mg), and trace elements become apparent [2]. There are many avenues still to explore for investigating the interactions of Fe with other elements during absorption and distribution. The impacts on different organs still need exploration. In this review, we refer to elements Fe, calcium (Ca), and magnesium (Mg), and trace elements according to the definition of Thomas [3].

Adequate intake of Ca, Mg, and trace elements, including copper (Cu) and zinc (Zn), is essential for human health [4]. These elements not only support basic physiological functions but offer insights into disease states when measured in diagnostic settings. Some of these non-essential elements can pose significant health risks due to their interference with Fe regulatory mechanisms when they are present in the body. For instance, lead (Pb), cadmium (Cd), mercury (Hg), and nickel (Ni) which are categorized as non-essential trace elements. These elements can enter the body through occupational exposure or environmental contaminants including factors influenced by lifestyle choices such as diet and smoking [5]. Understanding the impact of these trace elements on Fe metabolism and overall health is crucial for developing effective strategies to mitigate their adverse effects. However, extensive research on how trace elements interact and influence health is still lacking [6, 7].

The historical narrative surrounding Fe and its impact on human health is diverse with evidence for its significance dating back to ancient civilizations [8, 9]. Clinical manifestations of altered Fe homeostasis, e.g., changes in skin color, have been documented throughout history [8]. Notably, the influence of Fe on health and disease has been recognized since Egyptian, Greek, and Roman times. In the seventeenth century, a condition described as “green disease” (chlorosis) was treated with Fe supplements [9]. During the nineteenth century, a common ailment affecting young women was described as being characterized by lethargy, decreased work capacity, paleness, and amenorrhea. Interestingly, young women working in copper factories did not develop chlorosis. With contemporary knowledge regarding the interplay between Fe and Cu, exposure to Cu-salts in the workplace seems to have positively influenced Fe homeostasis. Nevertheless, distinguishing Cu deficiency from Fe deficiency anemia can be challenging [8].

Understanding of the essential role of Fe in hemoglobin synthesis and oxygen transport began to emerge in the early twentieth century. In the same century, a condition named “bronze diabetes” was described, in diabetic patients with pronounced skin pigmentation [10]. Hemochromatosis (from the Greek words “blood & color”) was described in 1865 by a French physician. The association of its phenotype with Fe overload was established in 1890 by a German pathologist, deepening the understanding of the relationship between Fe and health [11].

This review aims to evaluate the biological interaction with Fe of Ca, Mg, and selected trace elements (Cu, Zn, Pb, Cd, Hg, and Ni) and their roles in certain diseases. Unraveling the complexities of these interactions may enhance the understanding of how they affect human health.

## Iron

### Biochemical Properties

Fe is an essential transitional element in biological systems and exists in two oxidative states: (1) ferric (Fe^3+^) ion acting as an electron acceptor; (2) ferrous (Fe^2+^) ion, acting as an electron donor. This flexibility renders Fe pivotal in catalyzing redox reactions acting as a cofactor in numerous enzymes. Examples include Fe-sulfur clusters and heme groups found in hemoglobin, myoglobin, cytochrome, myeloperoxidase, and nitric oxide synthetases [12]. Consequently, Fe plays an indispensable role in fundamental cellular functions and overall biological processes. These encompass oxygen transport, aerobic respiration, intermediary and xenobiotic metabolism, nucleic acids replication and repair, innate and acquired immunity, and cell signaling pathways. Fe is predominantly chaperoned by proteins, enabling cells to harness its benefits while mitigating its potential harmful effects [13].

The average total amount of Fe in the human body is 40–50 mg/kg in adults, with the majority incorporated into hemoglobin (approximately 2.5 g). The remaining Fe is stored in ferritin, primarily located in the liver, bone marrow, and spleen. Humans efficiently conserve and recycle Fe primarily through erythrophagocytosis in reticuloendothelial macrophages with a recycling rate exceeding 80% [14, 15]. Both Fe deficiency and excess are associated with significant pathophysiological conditions. Fe balance is tightly controlled at a systemic level through the regulation of bioavailable Fe absorption. Unlike other elements, there is no physiological regulatory mechanism for excreting Fe. Intestinal absorption of bioavailable Fe determines Fe concentration in the body, with all unabsorbed Fe remaining in the intestine and potentially contributing to oncogenic processes. The amount of Fe in feces is ten times higher than in most tissues [16]. In this regulation process, several proteins play key roles. For instance, Trf binds Fe^3+^ to reduce its toxicity and facilitate blood transport while ferritin serves as a storage center and can release Fe when it is needed in the organism.

### Intestinal Absorption

Absorption of dietary Fe is a variable and dynamic process and serves as an initial point for the regulation of Fe balance. Normally, 1–2 mg of Fe is absorbed daily from the diet in the duodenum, and an equal amount of Fe is lost through shedding of gastrointestinal cells and epithelial desquamation. In general, humans absorb only 5 to 35% of ingested Fe, depending on various interactions between food compounds and the source of Fe. Bioavailable Fe refers to the portion of Fe absorbed from the diet that is accessible for use by cells in the body [2, 13, 17–19].

**Fig. 1:**
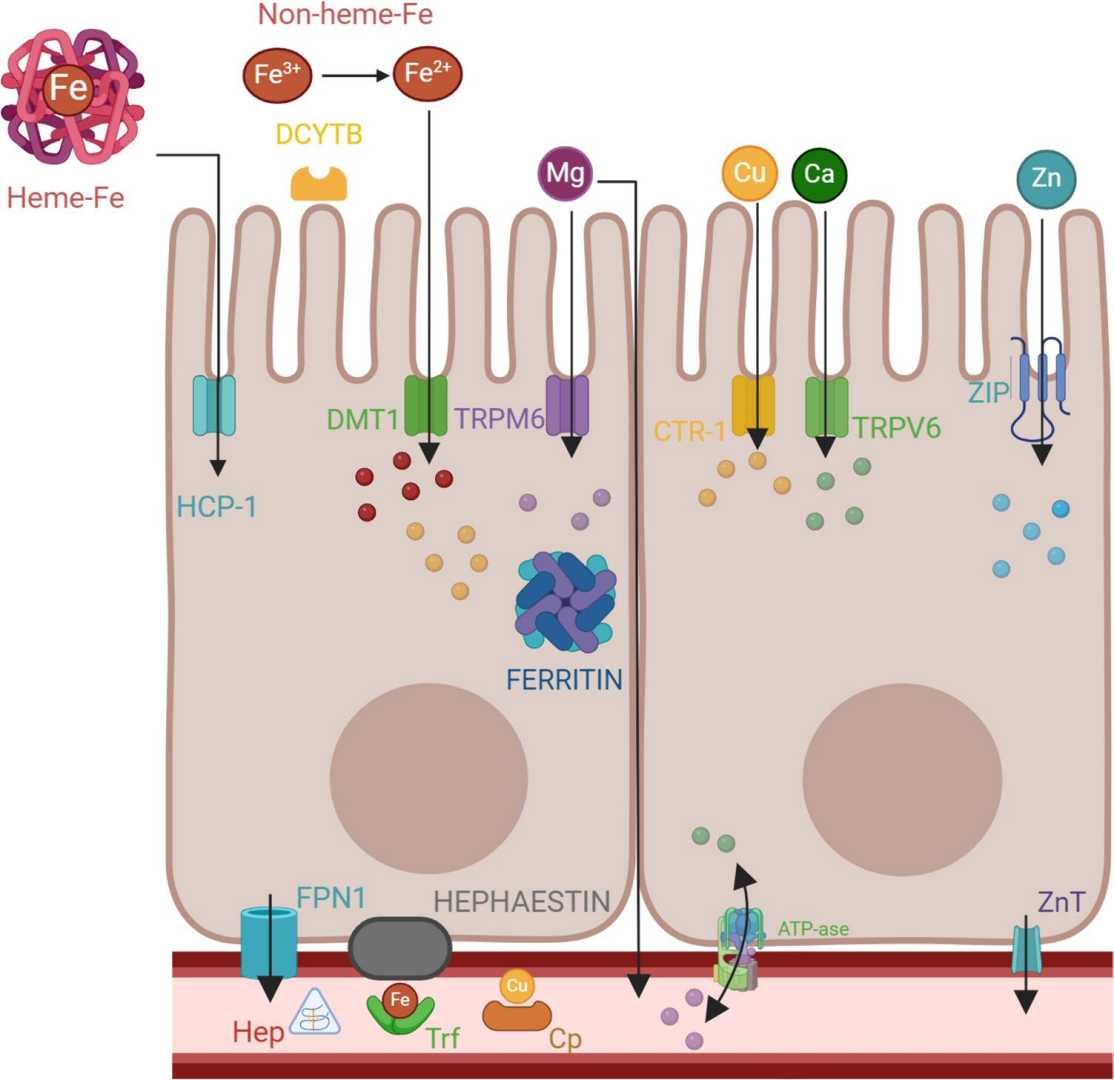
The absorption process of iron (Fe) at the enterocyte level. Heme-Fe is absorbed via the hem carrier protein 1 (HCP-1), while non-heme Fe is absorbed through the divalent metal transporter 1 (DMT1) after conversion of Fe^3+^ to Fe^2+^ by duodenal cytochrome B (DCYTB). Fe can either be stored in ferritin or exported into the bloodstream by ferroportin (FPN1), the sole known Fe-exporter, which is regulated by Hepcidin (Hep) through downregulation. Hephaestin and ceruloplasmin (Cp) facilitate the oxidation of Fe^2+^ to Fe^3+^ for binding to transferrin (Trf). Cells dependent on Fe have transferrin receptors on their surface, allowing them to uptake Fe from Trf. Cp also binds copper (Cu) and transports it to cells. Cu enters the enterocyte via the Copper transporter 1 (CRT1). Magnesium (Mg) and calcium (Ca) enter the enterocyte through specific transporters (TRPM6 and TRPV6), and both elements are exported into the blood through ATP-ase activity. Zinc (Zn) is imported via ZIP transporter(s), while efflux is via Zn transporter (ZnT) (created with BioRender.com)

Heme Fe (predominantly from animal sources) is considered the most available source of Fe with an absorption rate of 10–70%. Mechanistically, heme Fe is transmitted across the apical membrane through heme carrier protein 1 (HCP1) into enterocytes, where heme oxygenase releases Fe while catabolizing heme to biliverdin [19]. Non-heme Fe (mostly derived from plants) has an absorption ratio of 10–20% [20]. Its absorption is limited to the duodenum, whilst heme-Fe can be absorbed in other parts of the intestine [21]. Non-heme Fe is primarily in the ferric form, posing an entry obstacle that is overcome by reduction to Fe^2+^ by Fe reductase (DCYTB), influenced by factors such as pH (Fig. 1). The Fe^2+^ then enters the enterocyte through DMT1 or Zrt-Irt-like protein 14 (ZIP14) at the apical membrane.

Upon entry to cells, Fe can be used for protein and nucleotide synthesis, mitochondrial pathways regulating transcription via iron responsive element (IRE) binding proteins, stored in ferritin or released into the blood. The export of Fe is mediated by FPN1, often aided by hephaestin or Cp. Hephaestin is a ferroxidase that oxidizes Fe^2+^ to Fe^3+^ after Fe passes the basolateral membrane to bind Trf [21]. Once bound to Trf, Fe can enter the blood and be delivered to all cells, primarily through endocytosis of the transferrin receptor (TrfR 1/2). Ferroportin is the only known Fe exporter and is critical in regulating Fe homeostasis at the cellular level by post-translational regulation [21, 22].

If Fe storage is replete in the body, the liver will produce Hep which is a key player in controlling Fe absorption from absorptive enterocytes, Fe-recycling macrophages, and hepatocytes. Hep binds to FPN1 and induces its internalization and degradation in lysosomes, resulting in all absorbed Fe remaining in the cell and being unavailable for circulation. This is a defensive and immune response of the body to conserve Fe for physiological needs and make it unavailable for microorganisms. Several factors can stimulate or inhibit this process and errors in this regulation can lead to Fe deficiency or Fe overload and be associated by Fe-related diseases [22, 23]. Hep also downregulates both TfR1 and DMT1 through a yet unknown mechanism [24]. Thus, higher Hep expression inhibits dietary Fe absorption and Fe release from recycling macrophages and other body stores [14]. In contrast, a decrease in Hep concentrations promotes Fe availability. The interaction between Hep and FPN1 is one aspect of system regulation, other aspects include cytosolic iron-responsible and regulatory proteins (IRP-IRE), hypoxia-responsive pathways (HIF), and erythropoietin signaling. These types of Fe regulation are beyond the scope of this review and can be found elsewhere [11, 13, 25].

### Metabolism and Homeostasis

While all cells have the capability to import, export, or store Fe, certain cells are specialized for specific Fe-related functions. These include erythroblasts for Fe uptake, enterocytes and macrophages for Fe export, and hepatocytes for Fe storage [14]. Fe metabolism and homeostasis exemplify a complex circular economy governed by dietary absorption and regulated by hormones, the expression of relevant proteins and Fe discharge from macrophages. Anemia is a significant global public health concern affecting approximately one-third of the world’s population [26]. Children, adolescents, women of reproductive age, and the elderly are particularly vulnerable. Diagnosis of anemia relies on measuring hemoglobin concentration, using cut-off values < 130 g/L for males and < 120 g/L for non-pregnant females, though these thresholds are subject to debate due to ethnic differences [26]. Fe deficiency ranks among the top three causes of anemia worldwide, contributing to up to 50% of all cases. Deficiencies in vitamins, Cu and Mg can also lead to anemia due to their specific roles in hemoglobin and red blood cell production. Valuable insights can be obtained by exploring the interplay of elements including Zn, Cu, Mg, and Ca in anemia. Studies have found deficiencies in these elements in anemic children compared to non-anemic individuals [27]. Serum ferritin concentration is the most commonly used parameter to diagnose Fe deficiency. However, ferritin concentration can be affected by obesity, inflammation, chronic alcohol intake, liver or kidney disease, and cancer. Furthermore, discrepancies in ferritin measurement methods may hinder result interpretation.

Despite the clinical significance of elements in Fe-related pathologies, new biomarkers such as soluble transferrin receptors (sTrfR), Hep, and non-transferrin bound iron (NTBI) are not routinely measured due to limited sensitivity, specificity, and standardization. Standardized sampling practices are important as serum Fe concentrations measured by spectrophotometric methods can vary up to 70% daily within individuals. [28]. Reduced Fe recycling from macrophages, as observed in inflammation, can lead to anemia. Fe deficiency initially presents clinically as anemia [1]. True Fe deficiency is defined by diminished total Fe stores but reduced circulating Fe concentration can cause anemia due to functional Fe deficiency. This impairs erythropoiesis despite adequate or elevated Fe stores [13]. Functional Fe deficiency is a hallmark of anemia of chronic disease or anemia of inflammation associated with many chronic conditions. Inflammatory cytokines such as interleukin-6 activate the JAK/STAT3 signal pathway leading to induction of Hep transcription [13, 19]. Consequently Hep reduces FPN1 expression, inhibiting dietary Fe absorption and Fe recycling in macrophages contributing to limited Fe bioavailability for microorganisms or oxidative stress in chronic inflammation [23, 29].

Iron deficiency is common in chronic kidney disease due to the decreased Fe absorption caused by elevated Hep concentrations. Increased serum Hep in kidney diseases is partly due to reduced clearance of Hep as indicated by the inverse correlation of Hep with estimated glomerular filtration rate. As chronic kidney disease progresses, the kidneys fail to produce sufficient erythropoietin. This means that Fe cannot be released from stores rapidly enough to meet the demands for erythropoiesis, leading to functional Fe deficiency [13, 30, 31]. Conversely, Fe overload often occurs when errors take place in the Hep-FPN1 axis leading to FPN1 resistance to Hep and causing hemochromatosis. Hereditary hemochromatosis is an autosomal recessive disorder characterized by excessive Fe absorption and tissue deposition, potentially leading to severe complications such as cirrhosis and liver cancer. Assessing body Fe stores is crucial and consistently elevated ferritin concentrations combined with Trf saturation over 45% are indicative of primarily hemochromatosis. Mutations in the Hep regulatory and transmembrane protease serine 6 (TMPRSS6) genes, result in Fe-refractory-Fe deficiency anemia due to the inability to suppress Hep production in the liver [13, 14, 31]. Individuals with this disease do not respond to oral Fe supplementation and only partially respond to parenteral Fe supplementation. This is because of Hep-mediated FPN1 degradation, which mobilizes Fe from parenteral preparations [13]. Hep regulates FPN1 expression in macrophages, it is upregulated by heme and downregulated by inflammatory cytokines [14, 22].

### The Interplay Between Iron and Other Elements

The dynamic interaction between Fe and other elements primarily occurs in the small intestine, with the duodenum and jejunum serving as crucial sites for absorption. The efficiency of element absorption is a complex process and it is influenced by various factors. These include the chemical form of the element, dietary composition, intestinal pH, the physiological condition of the individual, and the presence of other minerals, elements, inhibitors, and enhancers. The mechanisms involved in the absorption of elements are passive transport (e.g., Zn), active transport (e.g., Ca), or a combination of both. Research suggests that there may be competition from other elements for the intestinal Fe absorption pathway (Fig. 1). Notably Cu and Cd have been identified as significant inhibitors of Fe^2+^ uptake, whereas Hg and Pb do not cause such an effect [32].

Humans and animals have developed intricate mechanisms for both absorbing and excreting essential trace elements as well as Ca and Mg. Disruption in homeostasis resulting in inadequate balance (deficiency or excess) can profoundly affect human health. The interaction between Fe, Ca, and Mg with other trace elements is orchestrated by some key proteins, especially DMT1 and DCYTB. The former has been implicated in the intestinal transport of Fe, Cu, Zn, and Cd, whereas the latter is able to change the oxidation state of both Fe and Cu ions [19, 27, 33]. For example, a main player in Fe export FPN1 may be influenced by Cu. Cu is actively involved in the function of hephaestin, a Fe^2+^ oxidase [8, 19]. Understanding the nuanced roles of these proteins and the broader interaction between Fe, Ca, Mg, and trace elements is essential not only for advancing our understanding of human physiology but also for addressing public health concerns.

In non-anemic adolescents, Choi et al. found that Fe concentrations correlated inversely (negatively) with Pb and Cu. In contrast, Pb correlated positively with Zn and correlation with Fe concentration was not observed for Zn and Cd [34]. The absorption of both Mg and Ca was found to be closely related to Fe in β-thalassemic patients compared to a control group. Mg was found to be high in thalassemic patients, whilst Ca was lower compared to the control group [35]. These three elements can compete for absorption in the intestinal, especially when they are consumed in large quantities. Whilst consumption of Ca has a reciprocal relationship with Mg absorption, the mechanism by which they interact individually or synergistically with Fe metabolism is still not fully understood [36]. Bolann and coauthors in their study showed that venipuncture in hemochromatosis patients led to changes in trace element metabolism, including increased the absorption of potentially toxic elements [37].

## Essential Elements: Copper, Zinc, Calcium, Magnesium

Cu and Fe share some physicochemical properties and their interactions have been previously acknowledged [8]. Cu may positively impact the absorption, transport, and utilization of Fe, whilst Fe may antagonize Cu metabolism. When Fe stores are depleted, Cu is redistributed to crucial tissues involved in regulating Fe balance, such as the intestine, liver, and blood. Cu may enhance Fe transport in enterocytes and increase the synthesis of Cp in hepatocytes, which is a significant circulating ferroxidase capable of releasing Fe from stores. Aceruloplasminemia is a rare genetic disease characterized by a gene mutation that reduces or abolishes Cp production, leading to Fe accumulation in tissues [33]. Interestingly, Cu depletion impairs the biosynthesis of Cp resulting in a similar Fe overload phenotype seen in aceruloplasminemia. Cu influences DNA binding activity, hypoxia-inducing factor (HIF) binding and modulates intestinal Fe homeostasis [8]. The regulation of Cu metabolism responds to physiological demand in the organism. Metallothionein, a protein binding both Cu and Zn with a higher affinity for Cu, plays a role in this regulation process. Depleted Cu concentrations are common in conditions such as hemochromatosis or when taking high doses of Fe supplements [8]. Notably in cases of Fe deficiency, Cu promotes Fe absorption in the intestine however compared to intestinal Fe absorption, less is known about the regulation of Cu absorption.

Absorption of Cu starts in the duodenum where Cupric ion (Cu^2+^) from the diet needs to be reduced prior to absorption (to cuprous ion (Cu^+^)) by DYCTB and hephaestin, among other cupric reductases. Cu^+^ is then transported into enterocytes through specific transporters such as Cu transporter 1 (CTR1) [38]. In Fe depletion, DMT1 can transport Cu, especially when little or no competing Fe ions are available for DMT1 in the intestine when Cu becomes a plausible ligand for this transporter. In the liver, Cu can be utilized, stored (as metallothionein), or incorporated into Cp. Similar to Fe, Cu is transported to the liver by binding to proteins (e.g., albumin) or amino acids (e.g., histidine). Ceruloplasmin and hephaestin share significant homology to each other and both incorporate Cu into their active sites and possess the capability to oxidize Fe [19].

Deficiency of Cu leads to defective Fe absorption with concomitant impaired erythropoiesis. The impact of Cu on hemoglobin synthesis is unclear but it is assumed that Cu may facilitate Fe import or utilization in mitochondria [8]. Moreover, Cu stimulates the synthesis of the biologically active form of Hep (Hep-25), which exhibits a high affinity for Cu binding [39]. Cu treatment of hepatoma cells through transactivation of the Hep antimicrobial peptide gene (HAMP) showed changes in the activity and expression of DMT1 (including its internalization) after Hep signaling, similar to the regulation of Hep-FPN1 interaction [39]. Recently, the Cu-binding properties of Hep-25 were harnessed in a novel detection assay for human Hep in serum [40]. Additionally, Cu may boost the antimicrobial and bactericidal properties of Hep-25 and influence FPN1 [41]. A deficit of Cu increases Zn absorption, whilst high supplemental Zn intake hinders intestinal Cu absorption, which concomitantly could induce severe Cu deficiency [8, 34].

Zn is a cofactor of 300 enzymes and plays a crucial role in nucleic acid metabolism, cell replication, tissue repair, and growth [42]. It also influences production, storage, and secretion of hormones and is ubiquitously present in all tissues, with the highest concentrations found in muscle, bone, and liver [43]. Zn has a critical role in heme synthesis (α-aminolevulinic dehydratase) and erythropoiesis [44, 45]. Its absorption occurs across the intestinal lining through specific a Zn transporter family (ZIP) including ZIP4, ZIP5, and ZIP14 which has been shown to transport both Fe and Zn in hepatocytes [8, 13, 43]. Zn is not a substrate for DMT1, and intestinal interactions are mediated by non-DMT1 mechanism [43]. The body maintains a stable concentration of Zn across a broad spectrum of dietary intake, suggesting the presence of an effective homeostatic mechanism in the organism to maintain balance [46].

Zn is stored as metallothionein and proteins including albumin (60%), α2-macroglobulin, and Trf (10%) are responsible for transporting Zn in the blood [43]. Transferrin transports both Fe and Zn, meaning that excessive Fe can compete with Zn absorption in the intestine and that Zn concentration increases when Fe concentration decreases [47]. Zn deficiency is associated with both reduced Fe absorption and elevated accumulation of Fe in tissue [43]. In a case–control study, Abdelheim and co-authors found that Zn concentrations were lower in subjects with Fe deficiency compared to a healthy control group. The same study proposed joint supplementation of Zn and Fe, particularly in patients with severe gut epithelial dysfunction.

Biomarkers for Zn deficiency have not been established to date [43]. In vivo studies suggested the possible role of FPN1 in exporting Zn and of Trf in binding Zn. Interestingly, excess accumulation of Fe may reduce Zn absorption and high concentrations of Zn may reduce Fe absorption [8, 34, 47]. Experimental animal models and in vitro studies on Zn deficiency documented Fe deficiency anemia as well as distribution/accumulation of Fe in tissues via induction of DMT1 and FPN1 triggering Fe uptake. Moreover, cross-sectional studies in humans revealed a positive association between Zn and hemoglobin, red blood cell indices, and ferritin. Biomarkers of Fe were higher in Zn replete subjects compared to Zn-deficient subjects. It has been suggested that an underlying Zn deficiency could induce Fe deficiency by blocking intestinal absorption and Fe mobilization from tissues [43]. Although Zn deficiency alone does not result in anemia, it can coexist with Fe deficiency. Excess Zn intake interferes with Cu uptake and may result in a Cu deficiency that eventually leads to anemia [45].

Zn is a key modulator of Fe absorption and tissue distribution. Compromised Zn status leads to a reduction in pancreatic Zn content, which in turn reduces intestinal Fe absorption through decreased expression of DMT1 and FPN1. In terms of mechanisms, the overall impact of Zn deficiency is linked to the onset of Fe deficiency, caused by a combination of diminished absorption in the intestines and reduced mobilization of Fe from storage [43]. Additionally, Zn may influence Fe homeostasis by regulating Hep expression in the liver through activation of the hemojuvelin-bone morphogenetic-SMAD (HJV-BMP-SMAD) pathway. A Zn-dependent enzyme called matriptase-2 (TMPRSS6) regulates the levels of HJV by proteolytic degradation. It is hypothesized that inhibition of matriptase-2 activity under low Zn concentrations may induce Hep production [43]. Intestinal Zn absorption reduces intestinal absorption of Ca and Cu, and Ca, Fe, and Cu have a negative impact on Zn absorption [34, 42]. Some trace elements (e.g., Pb and Zn) may share the intestinal Fe absorption pathway [2].

Ca is absorbed in the small intestine through a transcellular active transport process mediated by Ca channels and an electrochemical gradient, as well as through a passive paracellular process facilitated by tight junctions between enterocytes [8, 48, 49] (Fig. 1). Ca channels play an essential role in maintaining the steady state intracellular level of Ca. Despite being a divalent ion, Ca is absorbed by an independent cellular mechanism (TRPV6) and it is not an appropriate ligand for DMT1 [49]. Numerous studies have investigated the effect of Ca on Fe absorption, revealing a Ca dose-dependent relationship. This is because Ca is the only element capable of inhibiting both heme and non-heme Fe absorption, while other inhibitors of Fe affect only non-heme Fe absorption [2, 49–51]. The negative effect of Ca on Fe absorption has been examined in single and multiple meal studies but experimental and epidemiological data from these studies have not always been consistent, primarily due to study heterogeneity.

In their review and meta-analysis, Abioye and co-authors concluded that Ca supplementation in the short term has a negative effect on Fe status but the magnitude of this effect is unlikely to be biologically significant [50]. Two key mechanisms of action have been proposed for Ca and Fe interactions: (1) the luminal Ca internalization of DMT1 and (2) the interference of Ca with the transfer of Fe across the enterocyte basolateral membrane [49, 50]. Interestingly, higher Ca intake had no impact on hemoglobin concentrations [50]. The inhibitory effect depends on the coexistence of Fe and Ca in the intestine during fasting, so if possible Ca and Fe supplements should be taken separately [49]. It remains unclear whether there is a threshold level of Ca beyond which it can exert inhibitory effects on Fe absorption. The specific value of the Ca threshold and the potential factors influencing it are still unknown. Nevertheless, a randomized controlled trial by Keum et al., concluded that high doses of Ca supplements could reduce the risk of adenomas progressing to colorectal cancer, and the effect would extend with continued Ca intake [52].

Mg is widely acknowledged as a cofactor for numerous enzymes. Its critical role in hemoglobin synthesis means that deficiency in Mg can disrupt erythrocyte energy metabolism and the inflammatory process, leading to the development of anemia. This concept was further supported by evidence showing that Mg supplementation improved anemia in thalassemic mice and diminished erythrocyte membrane transport abnormalities in patients with sickle cell disease [53]. Mg is known to be an important coenzyme of glutathione peroxidase involved in hemoglobin synthesis based on experiments on animals [54]. In the intestine, Mg is absorbed through specific transporters like TRPM6 (transient receptor potential cation channel subfamily M member 6) or through passive non-specific diffusion between cells (Fig. 1). Intake of Mg is inversely associated with inflammation, and decreased Mg concentrations in serum correlate with inflammation, increased production of free radicals, and decreased Fe serum concentrations. In a cross sectional study among healthy Chinese adults, intake of Mg was inversely associated with risk of anemia. The mechanism by which Mg modifies Fe status is unknown however a lower risk of anemia was observed in individuals with highest Fe and Mg intakes suggesting Fe and Mg may jointly affect the risk of anemia [55].

Animal models also demonstrated that Fe deficiency increased the intestinal absorption of Mg and Ca [56, 57]. In athletes, hemoglobin concentrations and red blood cell count increased following consumption of Mg supplements [53]. Deficiency of Fe can lead to an increase in intestinal Mg absorption and certain Mg salts can raise pH or bind Fe and therefore negatively affect intestinal Fe absorption [53, 58]. In an interventional study conducted among female students, Milinkovic and co-authors suggested that short-term supplementation with Mg could have a beneficial effect on Fe biomarkers such as transferrin saturation [53]. An in vitro study showed that Fe absorption can be inhibited by Mg, however, the combination of Fe supplements with Mg was not considered a clinical problem. In a case report, Mg overuse led to Fe deficiency anemia which was refractory to long-term Fe supplements [59]. Mg deficiency has been shown to be closely related to a higher rate of anemia occurrence, especially among women and older subjects [54].

## Non-essential Trace Elements: Lead, Cadmium, Mercury, Nickel

Non-essential, xenobiotic, or toxic elements are commonly present in detectable concentrations within the human body. They typically infiltrate organisms by mimicking essential elements and exploiting their inherent transport systems. These elements gain entry through environmental, occupational, or intentional exposure, often resulting in toxicity and the development of pathological conditions [60, 61].

Pb present in measurable concentrations in the human body typically results from dietary ingestion. Plants have the ability to absorb Pb from contaminated soil and water (with regional variation); Pb can then transfer from plants to animals and humans. Pb is a harmful trace element and can affect Fe metabolism, especially in Fe deficiency disease. In children, Pb can lead to anemia and impair cognitive development [2, 62]. Upon absorption in the intestine via DMT1, Pb inhibits Fe uptake and can also interfere with Fe-dependent metabolic processes such as heme biosynthesis [27]. Fe deficit increases the absorption of Pb, whereas a high dietary Fe reduces Pb absorption in the intestine [27]. A study by Yazdani et al. found that Pb concentrations in hemochromatosis patients were raised after venipuncture therapy [63]. Similarly, another study involving hemochromatosis patients revealed increased Cd concentrations after venipuncture, compared to healthy subjects [64].

Cd is among the most widespread toxic elements. With properties similar to Ca, Cd can disrupt metabolism, leading to elevated Ca concentrations in urine. Cd has similar properties to Ca and therefore can disrupt its metabolism and cause elevated Ca concentration in urine. Cd toxicity is increased in the presence of Ca deficiency and Cd absorption also reduces concentrations of Fe, Cu, and Zn [42]. One well-known characteristic of Cd is its accumulation in the kidneys, where its long half-life causes it to act as a potential. Smokers in particular, are exposed to Cd due to its presence in tobacco plants that, directly absorb Cd from the soil [65]. Animal studies suggest that Cd decreases Fe concentrations by suppressing the expression of DMT1, FPN1, DCYTB, and Hep at mRNA level [42]. While Cd may inhibit Fe absorption in the intestine, the interactions between Cd and Fe remain unclear. In the case of Fe deficiency where DMT1 and FPN1 are upregulated, Cd absorption is increased [62]. Furthermore, Cd can induce various forms of anemia: (1) Fe deficiency anemia by competing with duodenal Fe absorption; (2) hemolytic anemia by deforming peripheral blood cells; (3) renal induced anemia by reducing erythropoietin production. Although Cd is able to bind to DMT1, this protein is not the sole transporter of Cd in the intestine. In a study using Caco-2 cells (a type of colorectal carcinoma cell line), Cd was found to inhibit the expression of the DMT1 gene, leading to decreased Fe concentrations in serum and resulting in anemia by suppressing Fe transport in the intestine. Cd may also inhibit genes related to the transport of both non-heme and heme Fe [66].

Similar to Cd, Hg serves no physiological function in the body. However, both elements have a high affinity for protein sulfhydryl groups and can be readily transported in the bloodstream [42].

Environmental Hg contamination primarily affects fish, shellfish, and other seafood, making dietary intake a common route of exposure and potentially leading to carcinogenic effects in humans. Methylmercury, is efficiently absorbed by the intestine (up to 95%) and predominantly bound to hemoglobin (> 90%), it can readily traverse the blood–brain barrier and accumulate in the brain. Additionally, Hg can contribute to the generation of reactive oxygen species within cells. In a study by Zabinski and colleagues, workers exposed to Hg exhibited higher ferritin concentrations compared to control groups [67]. Consistent with these findings, a positive correlation between Hg and ferritin was observed by Barany et al. [62]. However, Iturri and colleagues concluded that Hg did not significantly inhibit Fe uptake in mouse models [32].

Ni is considered essential for proper function of some organisms as it is known to enhance hormonal activity and is involved in lipid metabolism [68]. However, epidemiological in vivo and in vitro studies have also demonstrated its carcinogenic effect [69]. Absorption can occur through environmental and occupational exposures via the respiratory tract, digestive system, and skin. Occupational exposure to Ni through inhalation in factory workers has been linked to higher rates of lung and nasal cancers [69]. Ni is transported into the body and cells through Ca channels and DMT1. It specifically impacts on Ca homeostasis and mimics a hypoxia response, eventually leading cells to undergo carcinogenic transformation [69]. Ni can prevent the binding of Fe to Trf and in addition to competing for Fe uptake, Ni may alter Fe metabolism by increasing Fe IRP-1 binding activity and reducing Fe availability at the intracellular level [70]. Untreated and unrecognized Fe deficiency anemia may result in heightened sensitivity to Ni toxicity [71]. As described in a case report by Divya et al. a patient’s symptoms of urticaria were correlated with the consumption of green leafy vegetables, chocolates and nuts (i.e., foods rich in Ni). After proper Fe therapy, urticaria reduced and hemoglobin concentrations improved in the patient [72].

## Conclusion and Future Perspectives

Iron, similar to “Janus”, the two-faced Roman god can be toxic as it is essential. Disturbed Fe homeostasis may result in long-term consequences. This review considers the complex interplay and regulation of Fe with Ca, Mg, and selected trace elements, reflecting on the importance of Fe in selected diseases. The intestinal absorption and regulatory mechanisms governing systematic Fe homeostasis in relation to other elements, especially divalent ions, remain insufficiently explained. Numerous proteins including DMT1 (which imports Fe^2+^, Cu, Pb, and Ni), Cp, Hep, hephaestin (Cu is important in the structure), and Trf (binds Fe^3+^, Ni, and Zn) play crucial roles in mediating interactions among elements. Disruption in the absorption or tissue distribution of one element can change the homeostasis of other(s). Anemia, hemochromatosis, kidney disease, and carcinogenesis are among the diseases associated with disrupted Fe homeostasis, yet descriptions of the involvement of other elements in these conditions are scarce. Deficiencies in Cu, Mg, and Zn can lead to anemia due to their specific roles in hemoglobin synthesis. Depletion of Cp results in the accumulation of Fe in tissues. Hemochromatosis patients, who can experience complications such as liver cirrhosis and cancer if untreated, can also display elevated Pb and Cd concentrations when treated with bloodlettings. In kidney diseases, elevated Hep reduces Fe absorption and contributes to the development of anemia of chronic disease. Cd exposure and possible accumulation may complicate kidney disease. The interplay of elements forms a vicious circle in the onset of various diseases, making it challenging to discern cause and effect. The interaction of Fe with other elements is of particular concern in vulnerable populations, such as children and adolescents exposed to Pb and women of reproductive age exposed to Cd. Accordingly, any imbalance in Fe concentration and resulting disease should be viewed as a complex mechanism involving other elements and proteins. Today, many laboratories can measure most of these elements and proteins and thus give a better insight into the causes of the Fe imbalance. With such a comprehensive approach, it would be possible to achieve more effective treatment of the patient with improved and longer-term results.

Future research should focus on a thorough understanding of the proteins involved in intestinal absorption and tissue distribution of elements (DMT1, FPN1, Cp, Hep, hephaestin, Trf, and ferritin) and their interaction. Considering the diverse binding affinities of different elements to proteins, diverse sample types (full blood, serum, plasma, and urine) should be differentiated when measuring protein and element concentrations. Additionally, the approach to therapy, whether through dietary intake or supplements, is crucial in the treatment of long-term anemic patients (suggesting Ca and Mg supplementation). This review looks at a given limited number of elements influencing Fe metabolism, possible Fe interactions with other not here mentioned elements is an interesting topic for future research.

## Abbreviations

DCYTB: Duodenal cytochrome B
DMT1: Divalent metal transporter 1
Ca: Calcium
Cd: Cadmium
Cp: Ceruloplasmin
CTR: Copper transporter
Cu: Copper
Fe: Iron
FPN1: Ferroportin
HAMP: Hepcidin antimicrobial peptide gene
HJV-BMP-SMAD: Hemojuvelin-bone morphogenetic-SMAD
HCP1: Heme carrier protein 1
Hep: Hepcidin
Hg: Mercury
HIF: Hypoxia-responsive pathway
IRE: Iron responsive element
IRP: Iron responsive protein
Mg: Magnesium
Ni: Nickel
NTBI: Non-transferrin bound iron
Pb: Lead
TMPRSS6: Transmembrane protease serine 6, matriptase-2
sTrfR: Soluble transferrin receptors
Trf: Transferrin
TrfR: Transferrin receptor
TRPM6: Transient receptor potential cation channel subfamily M member 6, magnesium channel
TRPV6: Transient receptor potential vanilloid-6, calcium channel
ZIP: Zrt-Irt-like protein
Zn: Zinc
ZnT: Zinc transporter
